# Geochemical Integrity of Wellbore Cements during Geological Hydrogen Storage

**DOI:** 10.1021/acs.estlett.3c00303

**Published:** 2023-06-25

**Authors:** Adnan Aftab, Aliakbar Hassanpouryouzband, Abby Martin, Jackie E. Kendrick, Eike M. Thaysen, Niklas Heinemann, James Utley, Mark Wilkinson, R. Stuart Haszeldine, Katriona Edlmann

**Affiliations:** † School of Geosciences, University of Edinburgh, Grant Institute, West Main Road, Edinburgh EH9 3FE, United Kingdom; ‡ Curtin University, Discipline of Petroleum Engineering, 26 Dick Perry Avenue, 6151 Kensington, Australia; § Department of Earth and Environmental Science, Ludwig Maximilian University, Theresienstrasse 41, 80333 Munich, Germany; ∥ Department of Geosciences, Institute of Environmental Assessment and Water Research (IDAEA), Severo Ochoa Excellence Center of the Spanish Council for Scientific Research (CSIC), Jordi Girona 18-26, 08034 Barcelona, Spain; ⊥ School of Environmental Sciences, University of Liverpool, 4 Brownlow Street, Liverpool L69 3GP, United Kingdom

**Keywords:** net zero, hydrogen storage, geological storage, cementing, depleted gas fields, salt caverns

## Abstract

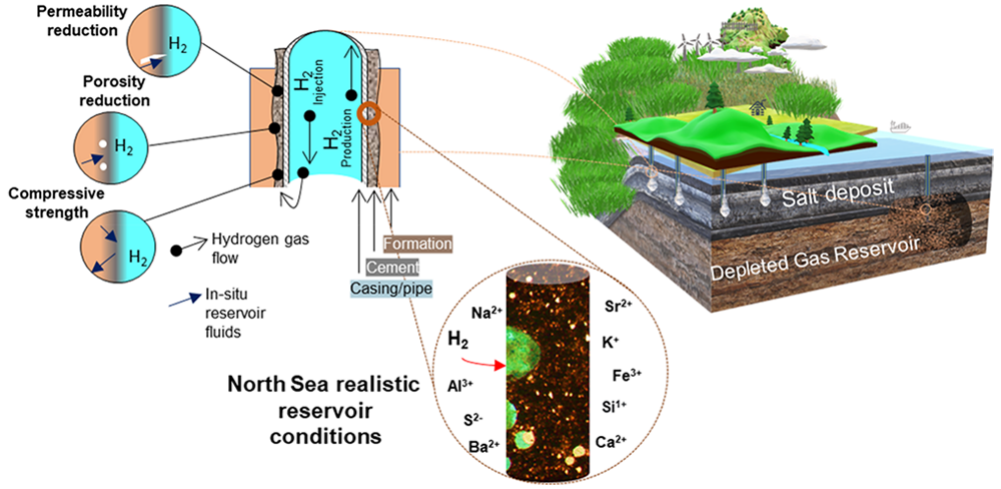

Increasing greenhouse gas emissions have put pressure on global economies to adopt strategies for climate-change mitigation. Large-scale geological hydrogen storage in salt caverns and porous rocks has the potential to achieve sustainable energy storage, contributing to the development of a low-carbon economy. During geological storage, hydrogen is injected and extracted through cemented and cased wells. In this context, well integrity and leakage risk must be assessed through in-depth investigations of the hydrogen–cement–rock physical and geochemical processes. There are significant scientific knowledge gaps pertaining to hydrogen–cement interactions, where chemical reactions among hydrogen, in situ reservoir fluids, and cement could degrade the well cement and put the integrity of the storage system at risk. Results from laboratory batch reaction experiments concerning the influence of hydrogen on cement samples under simulated reservoir conditions of North Sea fields, including temperature, pressure, and salinity, provided valuable insights into the integrity of cement for geological hydrogen storage. This work shows that, under the experimental conditions, hydrogen does not induce geochemical or structural alterations to the tested wellbore cements, a promising finding for secure hydrogen subsurface storage.

## Introduction

Climate protection agreements have been signed to curb global warming below 2 °C with the preference of achieving a target of 1.5 °C.^1^ Green hydrogen produced from electrolysis powered by excess renewable energy is a zero-carbon energy vector.^2,3^ Hydrogen has a higher gravimetric energy density (141.86 MJ/kg) compared to that of natural gas (55.5 MJ/kg). However, the volumetric energy density of hydrogen under standard atmospheric conditions is very low (0.0838 kg/m^3^) and requires either a high compression pressure of 70 MPa or a low liquification temperature of −253 °C for its storage at ground level.^4^ Geological storage is the leading option to provide the required volumetric capacity for grid-scale energy storage that can accommodate the supply and demand imbalances in the renewable energy sector at interseasonal time scales.^3^

**Figure 1:**
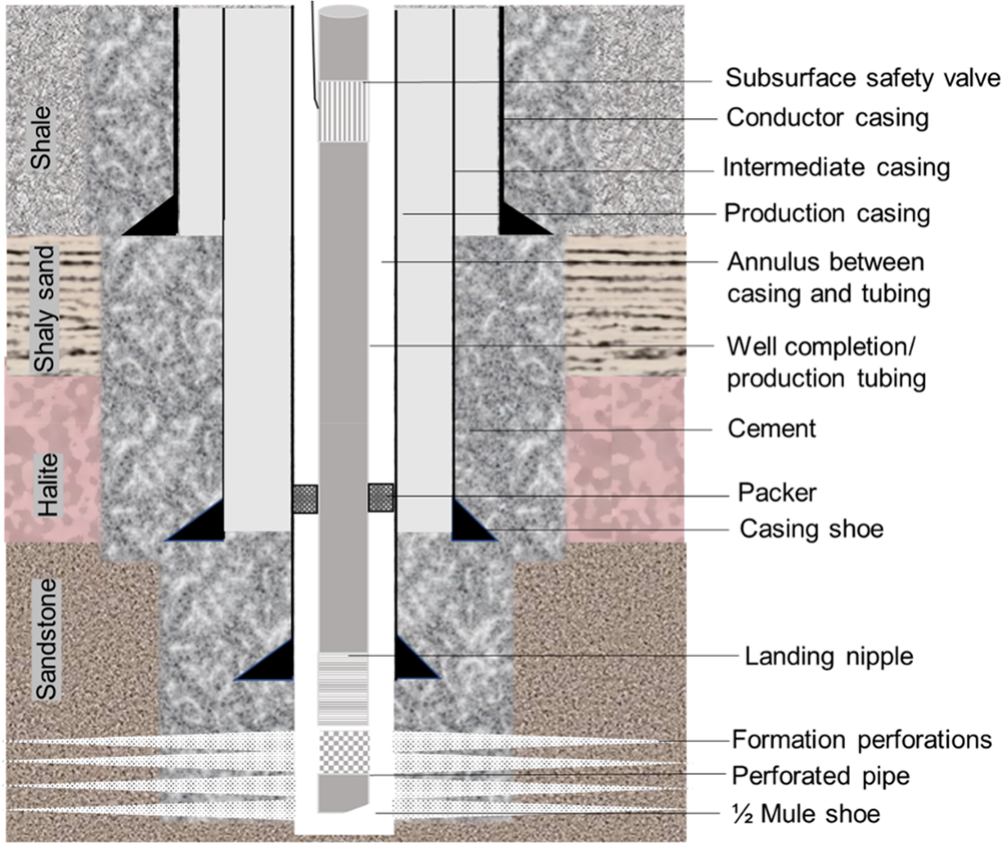
Schematic representing structural elements and cementing barriers associated with a typical well.

Salt caverns, depleted gas reservoirs, and saline aquifers offer promising hydrogen storage potential at a large scale.^5^ Experience with geological storage of natural gas has been developed over many decades, with more than 680 natural gas storage sites operational throughout the globe.^6^ In this regard, well integrity problems pose a risk to storage containment in a way that well integrity failure can lead to unintended leakage from the storage site during the life cycle of the well. As such, maintaining good integrity is critical to the long-term safe and efficient operation of any underground hydrogen storage site. A well contains a fixed set of structural elements (casing, cement, tubing, packers, and wellheads). Figure 1 illustrates multiple cement and casing barriers that simultaneously function to accomplish zonal isolation, which ensures the prevention of gas mixing or migration.^7^

Industry cements are predominantly manufactured in accordance with API Specification 10A standards. The API has identified eight different classes of cements that are deemed suitable for downhole conditions (well depth, pressure, and temperature), class A–H cements.^8^ Class G cement is the most used and preferred cement class, with 95% of industries worldwide opting for this class. Nearly all of these drilling cements are made of Portland cement, comprising lime (CaO), silica (SiO_2_), alumina (Al_2_O_3_), iron oxide (Fe_2_O_3_), and gypsum (CaSO_4_·2H_2_O). The raw materials undergo a complex series of hydration reactions that produce the four main compounds that make up Portland cement: tricalcium aluminate (Ca_3_Al_2_O_6_ or C_3_A), tricalcium silicate (Ca_3_SiO_5_ or C_3_S), tetracalcium aluminoferrite (Ca_4_Al_2_Fe_2_O_10_ or C_4_AF), and dicalcium silicate (Ca_2_SiO_4_ or C_2_S) (Table S1). Through curing, C_2_S, C_3_S, C_3_A, and C_4_AF are hydrated, and the hydration of C_2_S and C_3_S develops calcium silicate hydrate (3CaO·2SiO_2_·3H_2_O or CSH) and calcium hydroxide (Ca(OH)_2_ or CH), both of which promote early strength development aiding cementing integrity. Cement additives play a significant role, with more than 100 additives available to adjust the performance of cement specimens and tailor properties of cement according to the operational and site specific conditions.^9^ Hydrogen is a reducing agent and could potentially reduce oxidized additives that are present in the cements. Geochemical modeling shows that hydrogen can reduce the sulfate and ferric iron in cements to sulfides and ferrous iron, respectively, leading to the precipitation of oxide minerals and iron sulfide.^10^

However, there is a scarcity of key experimental data on the geochemical reaction of hydrogen with well cements. Herein, we report the results from laboratory batch reaction experiments concerning the influence of hydrogen on cement samples under simulated reservoir conditions and document valuable insights into the integrity of cement under hydrogen storage conditions to ensure a safe and secure operation process.

## Materials and Methods

**Figure 2:**
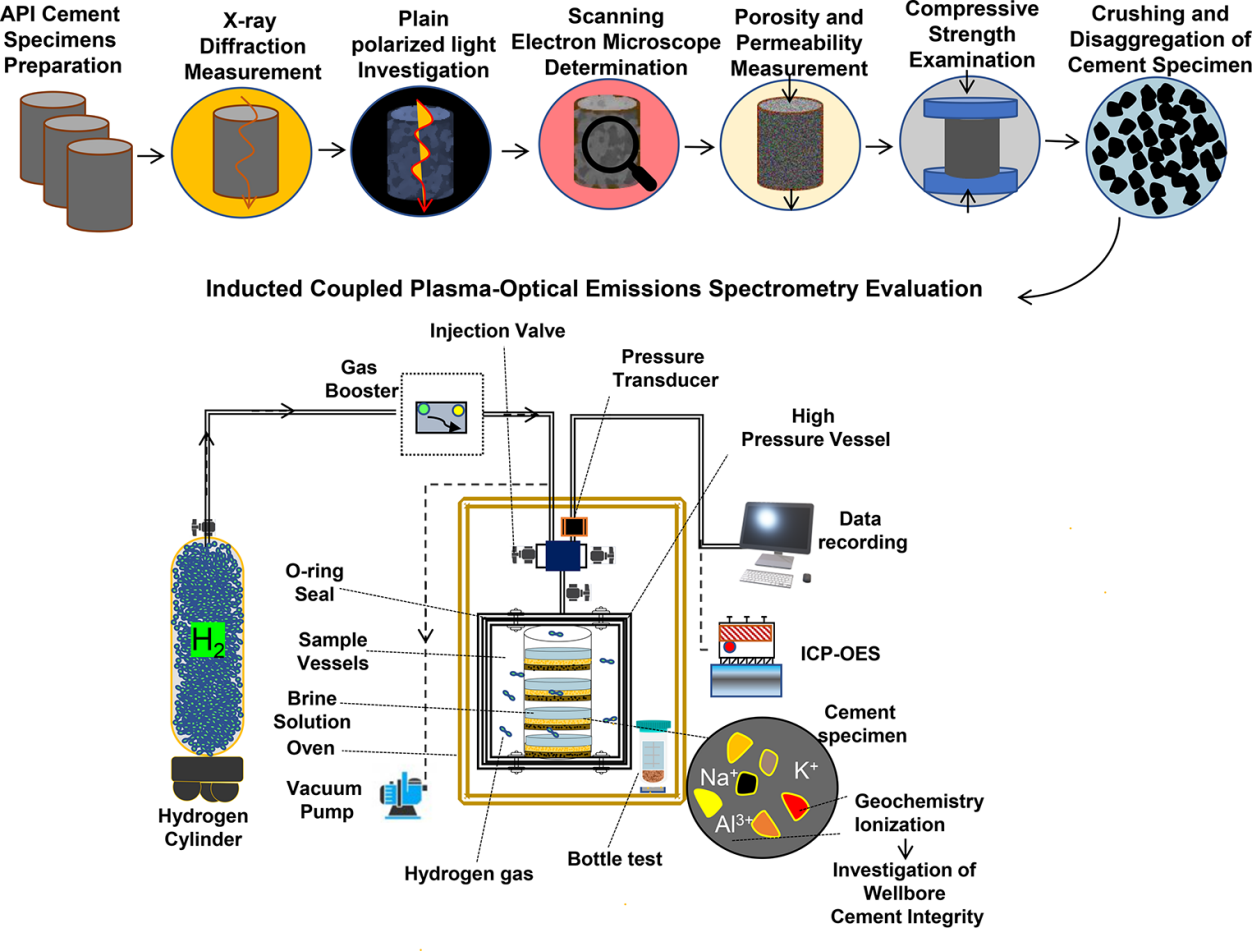
Methodology flowchart of this study that shows that first API cement samples were prepared. Then, the samples were characterized using XRD, plain polarized light (PPL) microscopy, and petrographic optical microscopy techniques. Finally, we determined the porosity and geochemical integrity of the specimen using the helium pycnometer and ICP-OES techniques.

The methodology has been devised with the aim of assessing the durability and integrity of well cements during underground hydrogen storage. Figure 2 illustrates a flowchart for the methodology used in this study. Experimental studies were initiated by preparing three cement slurries, including Portland cement (PC) without a retarder, PC with a retarder (i.e., AccuSET D197) (PCR), and Portland cement that was set under atmospheric conditions (PCA). For PC and PCR cements, a slurry was created at the Gerosion facility in Iceland by mixing a pre-prepared cement paste with water, with a cement:water ratio of 2.5:1. The cements were cured for 28 days in a humidity chamber; for the details of cement preparation, see the Supporting Information. The slurry for PCA was prepared with a cement:water ratio of 3:1 and air-dried for 36 h. This type of cement is representative of the material and composition that may be used for cementing wells and casing at a hydrogen storage site under API specifications.^11^

A range of analytical techniques were used to evaluate the materials used in the experiments, including X-ray diffraction (XRD), plane polarized light (PPL) optical microscopy, and porosity measurements (for details, see the Supporting Information).

To ensure that our experiments were representative of hydrogen storage conditions, we collated data from 138 depleted gas fields in the U.K. North Sea. A bar–whisker plot was constructed to reflect the maximum, minimum, mean, and upper and lower quartile information on temperature, pressure, and salinity^12^ (Figure S1). The chosen conditions are 3000 psi, 80 °C, and 35 and 250 ppt salinity. It is important to note that the conditions were limited by the experimental equipment; therefore, although they do not directly reflect a single field, they do comfortably sit within the averages across the North Sea.

The cement specimens were disaggregated and crushed manually with a pestle and mortar and then sieved to obtain a particle size of <355 μm. This was repeated for all 12 cement samples. Reservoir brine solutions were simulated by 35 and 250 ppt NaCl solutions in distilled water. The high-pressure experimental apparatus consisted of glass bottles set within cylindrical stainless steel batch reaction vessels sitting within a SciQuip-110S fan oven.

The disaggregated cement was then exposed to gas (hydrogen, nitrogen, or air) and brine under the simulated reservoir conditions. The experimental matrix is presented in Table 1. Table S2 lists the data for typical cement additives.

**Table 1:** Experimental Matrix Outlining the Conditions of the Experiments^a^

cement type	pressure (psi)	salinity (ppt)	container type	gas type	run time (days)
PC	3000	35	GSSV	H_2_	15.2
PCR	3000	35	GSSV	H_2_	15.2
PCA	3000	35	GSSV	H_2_	15.2
PC	3000	35	GSSV	N_2_	15.2
PCR	3000	35	GSSV	N_2_	15.2
PCA	3000	35	GSSV	N_2_	15.2
PC	atmospheric	35	PB	air	15.2
PCR	atmospheric	35	PB	air	15.2
PCA	atmospheric	35	PB	air	15.2
PC	3000	250	GSSV	H_2_	30.4
PC	3000	250	GSSV	N_2_	30.4
PC	atmospheric	250	PB	air	30.4

a PC, PCR, and PCA in glass in a stainless steel vessel (GSSV) and plastic bottles (PB). The specimens were tested at 80 °C. The quantity of each cement specimen was 15 g with a grain size of <0.355 mm in 50 g of water.

The sample fluid composition was determined both before and after the batch reactor experiments by using inductive coupled plasma-optical emissions spectrometry (ICP-OES). We used a Vista-Pro instrument with an Apex-E High Efficiency Inlet System. The ICP analysis indicates the elemental composition of brine/cement solutions with and without hydrogen interaction for 46 elements.^13^ Any value below the level of detection (LoD) was discounted.

For each sample to be analyzed, 6 mL of the brine solution was taken from each cement and combined with 1 mL of nitric acid. This acid is added to stabilize certain elements and to retain the elemental components within the solution. It is also an oxidizing agent, which assists in the breakdown of organic compounds better than other acids such as hydrochloric acid.^14^ A new syringe and filter were used for every sample to ensure no cross-contamination. The pH of the brines was taken before and after each batch reaction experiment and was determined using a METTLER TOLEDO Seven Excellence S500 pH Benchtop Meter with an InLab Micro Pro-ISM probe and an error range of <0.01. The detailed methodology for ICP analysis is provided in the Supporting Information. Further details about the methodology used for batch reaction experiments can be found in our recent publication.^3^

## Results and Discussion

### X-ray Diffraction Analysis

The mineralogy of PC, PCR, and PCA is relatively similar (Figure S2). All three cements contained quartz, calcite, and vaterite, with the PC, PCR, and PCA containing 52.6, 48.8, and 56.8 wt % quartz, respectively. The results show that the PCR slurry contains a higher percentage of vaterite (8.75%) than the PC (1.28%) and PCA (2.58%) slurries. Furthermore, the aragonite content is higher in the PCR (8.75%) and PCA (16.77%) slurries than in the PC (0%) slurry. The portlandite (PC, 17.95%; PCR, 15%) and larnite (PC, 12.82%; PCR, 7.5%) contents in the PC and PCR slurries were found to be higher than those of the PCA slurry, which lacked these minerals. Additionally, clinozoisite was detected in only the PC slurry, while gypsum (5.16%) and heulandite (1.29%) were present in only the PCA slurry. These mineralogical differences may affect the setting, hardening, and long-term durability of these cements.

### Optical Microscopy

Microscopic observations under PPL showed that the PC cement sample had the largest pores, which were irregular in size but had a high degree of sphericity and varied from 0.05 to 0.9 mm in diameter (Figure S3a). PCR has larger pores but a porosity similar to that of the PC sample within the uncertainty of the measurements (see Porosity of the Cement Samples, Figure S3b, and Table S3). PCA is dominated by pore sizes of <0.2 mm (Figure S3c). Note that these images represent the samples’ appearance prior to crushing in preparation for batch reaction experiments.

The cement minerals were largely unidentifiable under microscopic observation except for quartz (Figure S3d). The hydration reactions that form the cement result in an entanglement of hydration products, which leads to an abundance of amorphous (noncrystalline) phases of a colloid size.^15^ Light microscope identification is therefore not suitable for identifying minerals of this small size.^16^

### Porosity of the Cement Samples

The porosity measurements showed that the PCA had the highest porosity (36.50%), followed by the PC (31.2%) and PCR cement (29.1%). Table S3 lists complete details of specimen dimensions, masses of specimens, and porosities in the Supporting Information. These measurements coupled with the optical observations suggest that the PCR cement had the lowest porosity, localized into large pores when compared to the PC cement, while the PCA cement had the highest porosity, with pores that were much smaller and more widely distributed (Figure S3). In the initial phase of hardening, the pores have a greater connectivity. As the water content decreases and the hydration reactions progress, 59 hydration products are formed, which then expand and occupy the pore spaces. This results in the reduction of pore connectivity and overall porosity with extended curing time.^17^ Note that these values represent the samples’ porosity prior to crushing in preparation for batch reaction experiments.

### Geochemical Reactions under the Influence of Hydrogen

We consider that it is very unlikely that brine/hydrogen/cement reactions can occur without a corresponding change in porewater chemistry, where the dissolution of existing minerals will increase the concentrations of the associated elements in the fluid, while the precipitation of new phases will alter the equilibrium composition of the porewater. As such, fluid chemistry analyses can determine if any changes have occurred within the brine/hydrogen/cement system and are used as an indicator of reactivity.^18^

**Figure 3:**
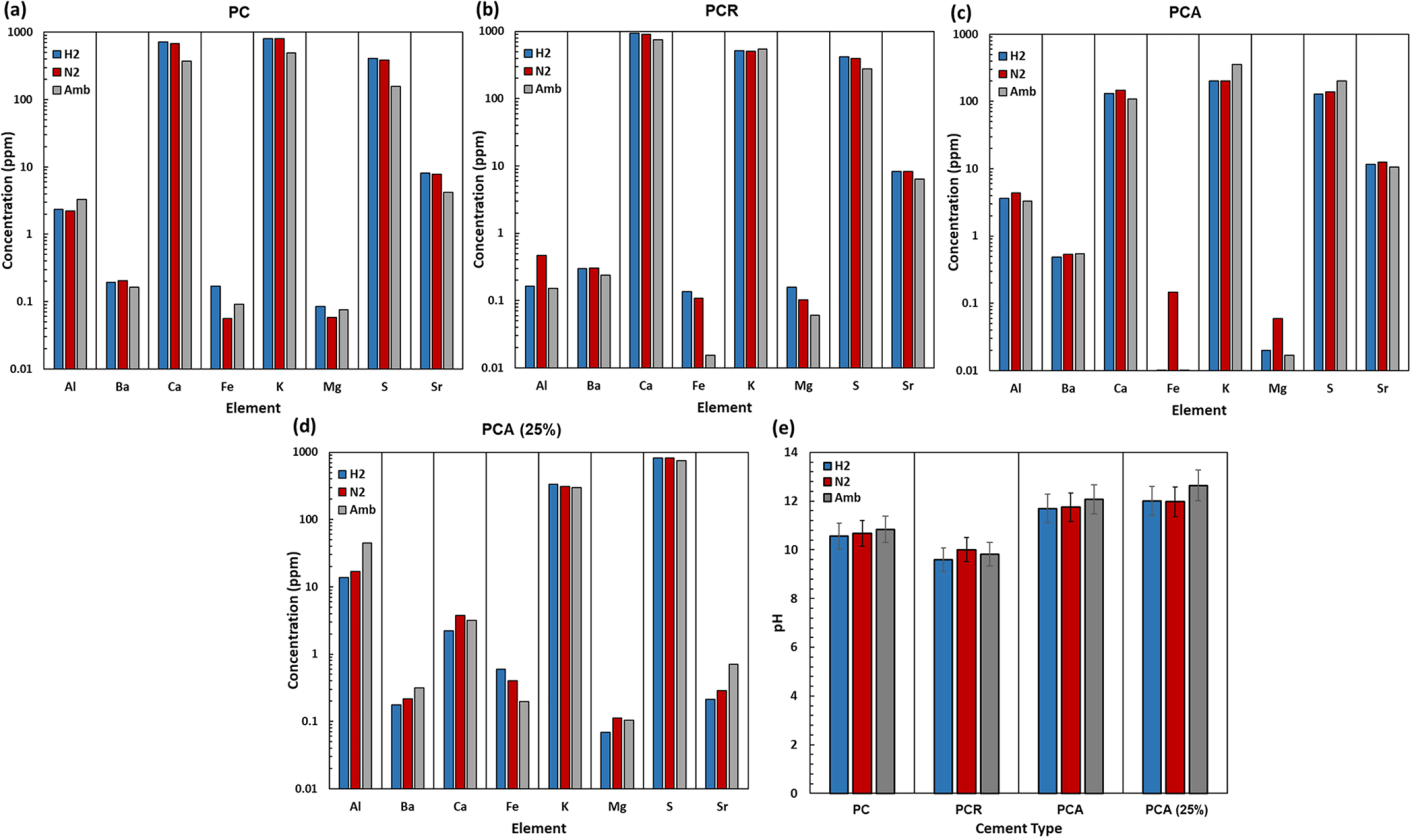
ICP results show the impact of hydrogen on (a) PC, (b) PCR, (c) PCA, and (d) PCA (salinity of 250 ppt; longer experimental duration) cement specimens against controls of nitrogen and ambient (atmospheric gas, Amb) at 80 °C and (e) pH values of brine after the experiments.

There were no significant changes in the elemental concentrations in the brine solution between hydrogen and nitrogen experiments, suggesting that no significant geochemical reactions occurred (Figure 3a–d). The main elements that relate to the reactions of concern for well cement integrity include sulfur (S), iron (Fe), and calcium (Ca). The change in the concentration of these elements could indicate reactions of hydrogen sulfide, pyrite, and calcium carbonate.^19^ As one can clearly see in the ICP results across all of the cement samples, there is minimal change to the concentrations of iron, sulfur, and calcium upon exposure to hydrogen when compared to nitrogen. The concentration of Fe is slightly reduced for PCA cement under H_2_ and ambient conditions (Figure 3c). The concentration of Fe in water and the occurrence of each oxidation state are controlled by pH, oxygen fugacity, and microorganism activity.^20^ Consequently, Fe might have been reduced in the presence of oxygen and high pH under ambient and H_2_ conditions in PCA, respectively. The minor changes observed in other elements do not extend beyond the natural variability within the cements and measurement repeatability and errors. Therefore, it is not indicative of any reactions taking place. It is worth noting that we investigated the repeatability error range of various elements in hydrogen batch reaction experiments in our previous article.^3^ These results indicate that no abiotic reactions occur in the tested well cements because of hydrogen within the experimental system after 2 weeks at a salinity of 35 ppt.

As shown in Figure 3e, the pH increases to >9 for all cements for hydrogen, nitrogen, and air and is constant within the uncertainty of measurement. The pH of PCR is lower than those of PC and PCA, plausibly explained by an acidic retarder, possibly hydroxycarboxylic acids.

A relationship exists among corrosion rates, microbial activity, and pH.^21^ In general, corrosion is more prone to occur in solutions with pH values of <7, and a pH of 5.5–9 favors microbial growth.^22^ The pH values for all cements indicate they do not sit within the favored ranges for corrosion or bacterial activity to occur (Figure 3e). Water in contact with Portland cement is expected to have a pH of 11–12.5, although some of the experimental values are below this range. This suggests that reactions between the cements and the brines (which are common to all of the tested gases) may not have reached equilibrium.^23^

The reactivity of PCA with hydrogen was further investigated at a high brine salinity of 250 ppt for one month, simulating the typical salinity of salt caverns,^24,25^ which may be appropriate hydrogen storage sites (Figure 3d). Again, the comparison of the elemental concentrations in the brine after hydrogen treatment with the concentrations in the controls showed a negligible change. Accordingly, the high salinity of 250 ppt does not affect the chemical reaction in the cement/hydrogen/brine system. It is worth noting that it is possible that chloride ions from the dissolved salts accelerate a hydration reaction mechanism leading to a reduction in the contact area between cement grains and water, and therefore, chemical reactions are less likely to occur in this system.^26,27^

Overall, this study showed that hydrogen does not react significantly with the three investigated wellbore cements. It therefore suggests a negligible impact of hydrogen on the integrity of the wellbore cements during geological hydrogen storage operations in salt caverns and porous rock reservoirs.

## Supporting Information Available

Fundamentals of well cement chemistry, cement additives, and assessment of hydrogen specific cements; methodology for factor selection, porosity determination, mineralogical data, petrographic optical microscopy, and fluid composition analysis; Portland cement reaction details (Table S1); common additives and functions (Table S2); details of porosity measurements on cement specimens (Table S3); pressure, temperature, and salinity data of 138 wells in North Sea fields (Figure S1); XRD of different cement specimens (Figure S2); optical microscopy images of different specimens (Figure S3); and safety measures (PDF)

## References

[ref1] Matos C. R.; Carneiro J. F.; Silva P. P. Overview of large-scale underground energy storage technologies for integration of renewable energies and criteria for reservoir identification. Journal of Energy Storage 2019, 21, 241–258. 10.1016/j.est.2018.11.023.

[ref2] Hassanpouryouzband A.; Joonaki E.; Edlmann K.; Haszeldine R. S. Offshore geological storage of hydrogen: Is this our best option to achieve net-zero?. ACS Energy Letters 2021, 6 (6), 2181–2186. 10.1021/acsenergylett.1c00845.

[ref3] Hassanpouryouzband A.; Adie K.; Cowen T.; Thaysen E. M.; Heinemann N.; Butler I. B.; Wilkinson M.; Edlmann K. Geological Hydrogen Storage: Geochemical Reactivity of Hydrogen with Sandstone Reservoirs. ACS Energy Letters 2022, 7, 2203–2210. 10.1021/acsenergylett.2c01024. 35844470 PMC9274762

[ref4] Aftab A.; Hassanpouryouzband A.; Xie Q.; Machuca L. L.; Sarmadivaleh M. Toward a Fundamental Understanding of Geological Hydrogen Storage. Ind. Eng. Chem. Res. 2022, 61 (9), 3233–3253. 10.1021/acs.iecr.1c04380.

[ref5] Groenenberg R.; Koornnef J.; Sijm J.; Janssen G.; Morales España G.; van Stralen J.; Hernandez-Serna R.; Smekens K.; Juez-Larre J.; Goncalves Machado C. Large-Scale Energy Storage in Salt Caverns and Depleted Fields (LSES)-Project Findings. 2020.

[ref6] Heinemann N.; Alcalde J.; Miocic J. M.; Hangx S. J.; Kallmeyer J.; Ostertag-Henning C.; Hassanpouryouzband A.; Thaysen E. M.; Strobel G. J.; Schmidt-Hattenberger C.; et al. Enabling large-scale hydrogen storage in porous media-the scientific challenges. Energy Environ. Sci. 2021, 14 (2), 853–864. 10.1039/D0EE03536J.

[ref7] Michanowicz D. R.; Buonocore J. J.; Rowland S. T.; Konschnik K. E.; Goho S. A.; Bernstein A. S. A national assessment of underground natural gas storage: identifying wells with designs likely vulnerable to a single-point-of-failure. Environmental Research Letters 2017, 12 (6), 064004 10.1088/1748-9326/aa7030.

[ref8] Parrott L. Effect of changes in UK cements upon strength and recommended curing times. Concrete (London) 1985, 19 (9), n/a.

[ref9] Broni-Bediako E.; Joel O.; Ofori-Sarpong G. Oil well cement additives: a review of the common types. Oil Gas Research 2016, 2 (1), 1–7. 10.4172/2472-0518.1000112.

[ref10] Jacquemet N.; Chiquet P.; Grauls A. In Hydrogen reactivity with (1) a well cement-PHREEQC geochemical thermodynamics calculations. 1st Geoscience & Engineering in Energy Transition Conference, 2020; European Association of Geoscientists & Engineers, 2020.

[ref11] Shi Z.; Jessen K.; Tsotsis T. T. Impacts of the subsurface storage of natural gas and hydrogen mixtures. Int. J. Hydrogen Energy 2020, 45 (15), 8757–8773. 10.1016/j.ijhydene.2020.01.044.

[ref12] Gluyas J. G.; Hichens H. M. UK oil and gas fields - an overview. Memoirs 2003, 20, 949–977. 10.1144/M52-2019-48.

[ref13] Balaram V. Rare earth elements: A review of applications, occurrence, exploration, analysis, recycling, and environmental impact. Geoscience Frontiers 2019, 10 (4), 1285–1303. 10.1016/j.gsf.2018.12.005.

[ref14] Grotti M.; Todolí J.-L. Nitric acid effect in inductively coupled plasma mass spectrometry: new insights on possible causes and correction. Journal of Analytical Atomic Spectrometry 2020, 35 (9), 1959–1968. 10.1039/D0JA00130A.

[ref15] Jones T. R. Metakaolin as a pozzolanic addition to concrete. Structure and Performance of Cements 2002, 372–398. 10.1201/9781482295016-21.

[ref16] Grutzeck M. W.; Roy D. M. Portland cement mineralogy. In Mineralogy; Springer US: Boston, 1983; pp 412–417.

[ref17] Zheng S.; Liu T.; Jiang G.; Fang C.; Qu B.; Gao P.; Li L.; Feng Y. Effects of Water-to-Cement Ratio on Pore Structure Evolution and Strength Development of Cement Slurry Based on HYMOSTRUC3D and Micro-CT. Applied Sciences 2021, 11 (7), 3063 10.3390/app11073063.

[ref18] Awadh S. M.; Al-Auweidy M. R.; Al-Yaseri A. A. Hydrochemistry as a tool for interpreting brine origin and chemical equilibrium in oilfields: Zubair reservoir southern Iraq case study. Appl. Water Sci. 2019, 9 (3), 1–12. 10.1007/s13201-019-0944-6.

[ref19] Laban M. Hydrogen Storage in Salt Caverns: Chemical modelling and analysis of large-scale hydrogen storage in underground salt caverns. Ph.D. Thesis, Delft University of Technology, Delft, The Netherlands, 2020.

[ref20] Pérez-Guzmán L.; Bogner K.; Lower B. Earth’s ferrous wheel. Nature Education Knowledge 2010, 3 (10), 32.

[ref21] Thaysen E. M.; McMahon S.; Strobel G. J.; Butler I. B.; Ngwenya B. T.; Heinemann N.; Wilkinson M.; Hassanpouryouzband A.; McDermott C. I.; Edlmann K. Estimating microbial growth and hydrogen consumption in hydrogen storage in porous media. Renewable and Sustainable Energy Reviews 2021, 151, 111481 10.1016/j.rser.2021.111481.

[ref22] Ismail M.; Md. Noor N.; Yahaya N.; Abdullah A.; Md. Rasol R.; A. Rashid A. S. Effect of pH and temperature on corrosion of steel subject to sulphate-reducing bacteria. J. Environ. Sci. Technol. 2014, 7, 209–217. 10.3923/jest.2014.209.217.

[ref23] Li Q.; Lim Y. M.; Flores K. M.; Kranjc K.; Jun Y.-S. Chemical reactions of portland cement with aqueous CO2 and their impacts on cement’s mechanical properties under geologic CO2 sequestration conditions. Environ. Sci. Technol. 2015, 49 (10), 6335–6343. 10.1021/es5063488. 25893278

[ref24] Ravikumar P.; Prakash K.; Somashekar R. Evaluation of water quality using geochemical modeling in the Bellary Nala Command area, Belgaum district, Karnataka State, India. Carbonates and Evaporites 2013, 28 (3), 365–381. 10.1007/s13146-012-0124-3.

[ref25] Dopffel N.; Jansen S.; Gerritse J. Microbial side effects of underground hydrogen storage-Knowledge gaps, risks and opportunities for successful implementation. Int. J. Hydrogen Energy 2021, 46 (12), 8594–8606. 10.1016/j.ijhydene.2020.12.058.

[ref26] Okhovat M. R.; Hassani K.; Rostami B.; Khosravi M. Experimental studies of CO2-brine-rock interaction effects on permeability alteration during CO2-EOR. Journal of Petroleum Exploration and Production Technology 2020, 10 (6), 2293–2301. 10.1007/s13202-020-00883-8.

[ref27] Kaushik S.; Islam S. Suitability of sea water for mixing structural concrete exposed to a marine environment. Cement and Concrete Composites 1995, 17 (3), 177–185. 10.1016/0958-9465(95)00015-5.

