# Supplementary material for: Geochemical Integrity of Wellbore Cements during Geological Hydrogen Storage
Source: Environ Sci Technol Lett. 2023 Jun 25;10(7):551–6. doi: 10.1021/acs.estlett.3c00303 (PMC10339721; doi:10.1021/acs.estlett.3c00303)
Supplement: Supplementary file 1 — ez3c00303_si_001.pdf [file ez3c00303_si_001.pdf]

Supplementary information

Geochemical integrity of wellbore cements during geological hydrogen storage

Adnan Aftab[a b], Aliakbar Hassanpouryouzband[a*], Abby Martin[a], Jackie E. Kendrick[a,c], Eike M. Thaysen[a,d], Niklas Heinemann[a], James Utley[e], Mark Wilkinson[a], R. Stuart Haszeldine[a], Katriona Edlmann[a*]

a) School of Geosciences, University of Edinburgh, Grant Institute, West Main Road, Edinburgh EH9 3FE, United Kingdom
b) Curtin University, Discipline of Petroleum Engineering, 26 Dick Perry Avenue, 6151 Kensington, Australia
c) Department of Earth and Environmental Science, Ludwig Maximilian University, Theresienstr. 41, 80333 Munich, Germany
d) Department of Geosciences, Institute of Environmental Assessment and Water Research (IDAEA), Severo Ochoa Excellence Center of the Spanish Council for Scientific Research (CSIC), Jordi Girona 18–26, 08034 Barcelona, Spain
e) School of Environmental Sciences, University of Liverpool, 4 Brownlow Street, Liverpool L69 3GP, United Kingdom

- **The geological storage well environment**

Underground gas storage (UGS) wells are very similar regarding design specifications to hydrocarbon production wells [1]. A well contains a fixed set of structural elements (casing, cement, tubing, packers, and wellheads) to create multiple barriers that simultaneously function to accomplish zonal isolation. Zonal isolation refers to the prevention of gas in one zone, from mixing or migrating to another zone [2]. Wells are commonly recognised as the most likely pathway for fluid migration; therefore, construction and efficient cementing practices are paramount to achieving the long-term durability of the well.

[*] Corresponding Authors: A.H: Hssnpr@ed.ac.uk, K.E: Katriona.edlmann@ed.ac.uk

The requirement of efficient and concise cementing of well operations has wider implications within the United Kingdom Continental Shelf (UKCS). Over 7,800 wells have been drilled in more than 430 hydrocarbon fields to date, with approximately 50% of these wells still in active operation [3]. In 2017, the Oil & Gas Authority (OGA) reported a production loss of 33 million barrels of oil equivalent (boa) across the UKCS. Upon investigation, this production loss was largely attributed to well failure. Well integrity accounted for 43.46% of this loss, with failure of the annulus accredited to 37.71% of this failure within the integrity issues[4]. This production loss shows the critical importance that proper cement and completion jobs have on ensuring the longevity of well operations.

For the geological storage of hydrogen to be successful, three requirements need to be met by the storage medium: storage capacity, injectivity and confinement. The last condition is crucial and primarily concerns the casing and cementing. Cementing has been continually evolving for over 100 years, with changing formulations, technologies, and materials. This development has been with the target in mind to withstand rigorous well operations and conditions, and to maintain that zonal isolation indefinitely [5]. Although 24 there is in-depth knowledge of the petroleum industries cementing requirements, there remains a lack of knowledge on cementing practices under hydrogen storage. The lack of active large-scale projects means the behaviour, long-term stability, and cement integrity is unknown [6].

- **Fundamentals of Well Cementing**

The principal goal of cementing a well is to retain and provide wellbore integrity. Wellbore integrity can be defined as the prevention of unintended fluid movement or loss during the lifecycle of a well, ensuring a long-term safe and efficient operation.

The assurance of wellbore integrity is associated with two phases: the construction phase (completion) and the plugging/remediation/abandonment phase [7]. The initial construction phase involves the placement of stainless-steel casing into a drilled hole and the installation of the cement. Well cementing comprises of adding a cement slurry into the annular (open) space between the well casing and surrounding rock formation [8]. Cementing a well involves two standard operations: the primary cementing and the remedial (secondary) cementing. Primary cementing is the initial introduction of a cement sheath into the annulus between the casing and formation. This sheath is considered critical to a well as it implements a hydraulic seal that initiates zonal isolation, restricting fluid migration between the borehole producing zones and preventing the leakage of gas to the surface. The sheath also acts as an anchor supporting the casing string and safeguards the casing from corrosion [9]. Multiple primary cementing jobs are often required as a well usually consists of numerous casing strings that individually need to be cemented. The remedial (secondary) cementing is when cement is strategically placed in certain well locations for purposes such as repair or well plugging/remediation/abandonment. The second phase involves the insertion of cement through the steel casing to shut in or abandon the well. All wells spanning across the UKCS and worldwide will eventually need to be plugged and abandoned once the reservoirs are depleted and operation has ceased. The objective of abandonment is to return the formations to its pre-drilling condition [10]. Well abandonment rates have increased four-fold since 2016, with 150 wells per year are expected to be plugged and abandoned going forward [3]. Consequently, this will account for more wells being plugged and abandoned than drilled. This adds additional stresses to the wellbore environment, making the durability of these materials over an extended period essential.

- **Well Cement chemistry**

Industry cements are predominantly manufactured in accordance with the American Petroleum Institute (API) Specification 10A standards. A controlled mixture of these compounds in adjusted proportions allows for a variety of cements to be made suited to different well conditions.

Table S1. Portland cement hydration reaction compound characteristics (data from reference[11]).

**Compound**	**Formula**	**Characteristic**
Tricalcium Aluminate	$Ca_3Al_2O_6$.	Releases lots of heat during initial hydration. Not used for strength properties. Addition of gypsum slows down hydration speed. Low ratio = more sulfate resistant.
Tricalcium Silicate	Ca_3SiO_5	Hydrates and hardens rapidly. Primarily liable for the initial set and quick strength gain.
Tetracalcium Aluminoferrite	$Ca_4Al_2Fe_2O_{10}$	Provides reduction of melting temperature in the kiln for raw materials. Results in rapid hydration, but not used for strength properties.
Dicalcium Silicate	Ca_2SiO_4	Hydrates and hardens slower. Used for onset of strength after one week.

- **Cement Additives**

Cement additives play a significant role within cementing operations and are classified by the API as materials that are added to a cement slurry to change or improve the cement properties and performance [12]. There is a large range of cement additives that have been developed to blend with base cement slurries and to be implemented in operations. An additive is selected and quantified pertaining to specific well conditions, where the appropriate cement slurry can be prepared for the well [12]. Each additive has a different functionality and designated purpose, as outlined in Table S2.

Table S2. Common additives, examples, and function. (Data from references[12, 13]).

**Additive Type**	**Additive Example**	**Properties**
Accelerator	Calcium chloride	• Speeds up hydration<br>• Reduces thickening time<br>• Early on-set strength
Dispersant	Polynapthalene Sulfonate	• Reduces friction to increase flow properties of the slurry<br>• Promotes slow growth of outer cement dimensions to allow cement to bond better to case and formation
**Expansion**	Calcium Oxide	
**Extender**	Bentonite	• Reduces slurry density

		• Increasing the slurry quantity to support long cement columns
Fluid Loss Agent	Hydroxethyl cellulose	• Deters dehydration of slurry's to prevent fluid through permeable strata
Retarder	Lignosulfonate	• Decreases hydration speed and water content<br>• Reduces thickening time and delays setting
Weighting Agent	Hematite	Restrains high formation pressures

- **An Assessment of Hydrogen Specific Cements**

One of the most important completion jobs within the well cementing industry is to design an appropriate cement mixture that considers a multitude of factors (downhole conditions) during the design period[8]. Studies have identified the primary reason of cement sheath degradation to be poor cement design[14]. Knowledge of a cement's particular behavioral parameters prior to the use of it in a well is key to ensuring the safety and durability of the operation. The design of a cement mixture under hydrogen storage conditions will need to exhibit the characteristics of gas tightness and chemical

resistance. Gas tightness is to ensure no gas escapes or penetrates through the cement to surrounding formations. Equally, chemical resistance is to provide durability against substances such as H_2S. This can be achieved by including corrosion inhibitors and shrinkage reducers to improve the chemical and corrosive properties [1]. Reducing the permeability by decreasing the water-cement ratio is a strategy often adopted to combat initial corrosion. By reducing the permeability, it reduces the available surface area absorption of corrosive chemicals. Other additives such as water reducing agents, fly ash, silica fumes and polymer admixtures like latex, also aid in reducing permeability[15]. Shrinkage reducers are recommended to avoid the initial mass shrinkage of the cement upon setting to decrease the likelihood of cracks forming and creating gas migration pathways. A study conducted by Vralstad et al in 2016[5], to identify the long-term durability of Portland well cement under downhole conditions, identified that including silica flour prolonged the durability of the cement significantly. The inclusion of this additive was found to be even more effective when exposed to corrosive conditions containing substances such as hydrogen sulfide (H_2S). This is particularly relevant where plugged and abandoned wells are concerned, as companies rely on the integrity of the cement to withstand downhole conditions[5]. It is worth noting that admixtures do not necessarily prevent or stop chemical attacks and corrosion, but they can slow the process significantly. The cements used in this study contain additives that one would expect to see used in hydrogen storage operations (silica flour, retarder). The suitability and combinations of admixtures are assumed to be what one would expect to use during hydrogen storage, to account for the potential of H_2S corrosion and gas migration pathways forming via cement cracking. This is a significant gap in knowledge that will be imperative to expand on if

large-scale hydrogen storage is to be implemented, as cement design is a fundamental factor of an operation.

Supplementary Materials and Methodology

- **Factor selection**

The experiments are based on twelve samples of three cement types; one Dyckerhoff Class G cement without retarder; one Dyckerhoff Class G cement with retarder; and one standardized Portland cement. These cements are hereafter referred to as PC, PCR and PCA.

- **Cement mix**

The cement design is based on a Dyckerhoff Class G cement with a combination of materials used to cast the hardened Portland cement, mixed with silica flour (40wt%). A slurry was created by mixing a pre-prepared cement paste with water, with a cement-water ratio of 2.5:1. Two types of hardened cement were prepared: one well cement without a retarder (referred to as PC) and one well cement with a Schlumberger AccuSET D197 retarder (referred to as PCR). The PC cement cured for a total of 28 days in a humidity chamber with 100% relative humidity at 20°C. The PCR sample was kept at 80°C and 100% relatively humidity for 2 days before being placed at the same condition as the PC cement (20°C and 100% relative humidity for 26 days). This curing method was adapted as the cement grout cannot harden at ambient temperature with the addition of the retarder, which delays the primary setting time of cement [16]. Cement sample three is a standard Polycell Polyfilla Quick Set cement, otherwise known as a standard Portland cement (referred to as PCA). The cement was air dried for 36 hours with a cement-water ratio of 3:1. This type of cement is

representative of the material and composition that may be used for cementing wells and casing at a hydrogen storage site under API specifications[17].

- **Porosity determination**

The porosity of the hardened cements was determined using an AccuPyc 1340 Helium-Pycnometer from Micromeritics. The device utilizes helium as a gas displacement method to measure the volume of the cylindrical cement samples[18]. In combination with the skeletal volume the measurement is used to calculate the fraction of connected pores [19]. The error range for the porosity measurements are in the vicinity of ±0.1% porosity.

- **Mineralogical analysis**

To characterise the composition of the cement samples, in terms of mineralogical assemblage and crystalline mineral phases, the samples are analysed by X-Ray Diffraction (XRD) using a Bruker D8-Advance X-Ray Diffractometer, which employs a 2-theta, Bragg-Bretano configuration. An XRD pattern is an outline of the intensity of X-Rays scattered at angles from 39 2-65°. This results in diffractograms which are then compared to the internal Bruker mineral database using the EVA analysis package. This database allows the detection of specific mineral 'phases' in which each phase is host to a unique diffraction pattern with distinct chemistry. Further analysis quantifying the amounts (wt%) of minerals present in the samples are used, otherwise known as Rietveld analysis[23]. This procedure gives a typical detection limit of 1wt%.

- **Petrographic optical microscopy**

Mineralogical analysis is employed using a Carl Zeiss Ultraphot III microscope, which has been upgraded for digital microscopy by the addition of a Canon 1100D DSLR camera. Thin sections are examined of the three cements pre-experiment.

Petrographic analysis of the cement provides unique information concerning the minerals, texture (sorting, grain size and grain contacts), framework grain composition and the distribution and type of porosity and permeability seen within the samples[24].

- **Simulated in-situ reservoir conditions**

Downhole reservoir conditions were simulated and controlled through geochemical batch reactions where the reactivity of hydrogen with cement was studied, using injection gases of hydrogen and nitrogen (of certified purities), reservoir brine (a mixture of sodium chloride and deionized water) and disaggregated cement samples. The controlled experimental parameters include the pressure, temperature, solution salinity, cement type, particle size and cement-water ratio. This control of individual parameters allows for the identification of contributing factors that change with the addition of hydrogen.

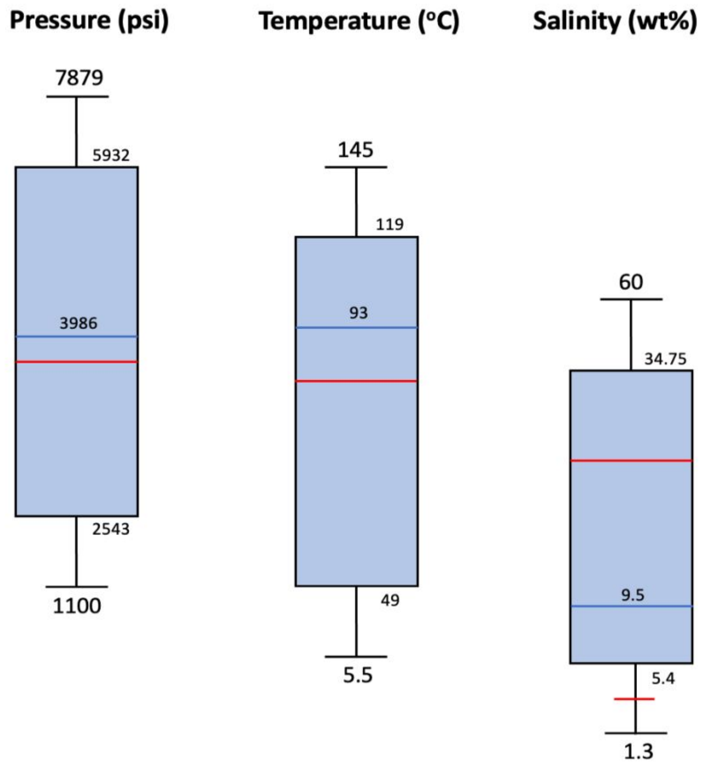

Figure S1 The maximum, minimum, median, lower, and upper quartile values for pressure, temperature, and salinities of 138 wells in North-Sea Fields. Red line shows the chosen experimental conditions and where they sit within the North Sea range.

- **Fluid composition analysis**

Glass bottles were used over stainless steel to prevent the contamination and damage from steel degradation and corrosion such as hydrogen embrittlement. Stainless steel was chosen as it is deemed a suitable material for high pressure vessels and the use of the oven keeps the experiment steady and resistant to the influence of external factors (i.e., ambient conditions) and to achieve elevated reservoir conditions in the cell. The vessel temperature and pressure were measured using a GD4200-USB Digital Pressure Transducer from Elemental Science Inc (ESI), with expected error of ±1.5%. The reaction vessels' pressure and temperature are constantly monitored via data transfer to a PC with ESI-USB software, which is then recorded using LabVIEW software by National Instruments. The oxygen within the vessels is removed via vacuum pump and nitrogen flow as a MTIG10-75-2 Gas Booster System with DLE75-2 inert gas booster is able to pressurise up to 10×103 psi. The injection gases of hydrogen and nitrogen are injected through a high-pressure valve at the top of the vessel.

To ensure continuity across the measurements, the samples were left to cool to room temperature after removal from the batch reaction vessel oven, and each sample tested a minimum of three times to allow for slight fluctuations and an equilibrium of the pH to be reached. Allowing an experiment to cool to room temperature can also adversely affect the measurements, as changes in temperature significantly impact pH and introduce variability[26]. To account for this, the METTLER TOLEDO has a built-

in calibrated system to account for the variation and correct it to within an error range. The pH was studied for any changes to give an indication of whether any reactions have occurred, for example the pH can change significantly if gasses such as H_2S or CO_2 dissolve in the reservoir fluid.

Supplementary Results

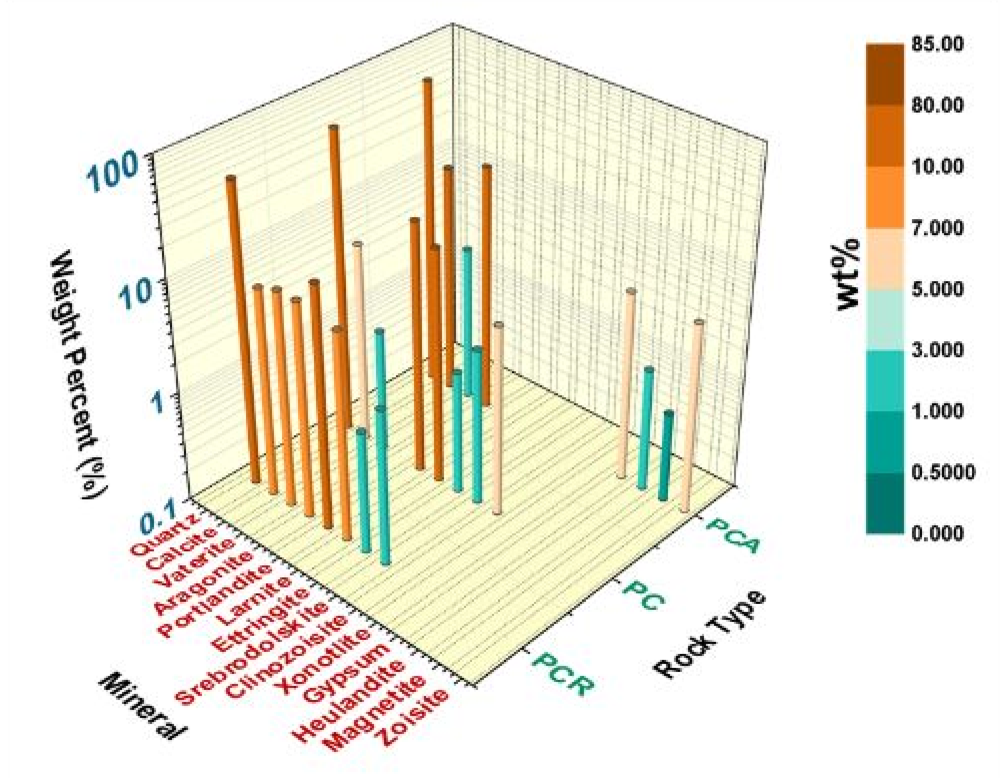

Figurre S2. XRD Minerological analysis of PC, PCR and PCA

Table S3 Details of different solid cylindrical cement specimens and porosity data

Cement type	Mass (g)	Length (mm)	Diameter (mm)	Length(cm)	Diameter (cm)	Length:diameter ratio	Bulk Volume (cm^3)	Calculated whole sample Density (g/cm^3)	Measured solid Volume (cm^3)	Measured solid Density (g/cm^3)	Open Porosity (%)	Average open porosity (%)
PC	10.95	12.36	25.86	1.24	2.59	0.48	6.49	1.69	4.47	2.45	31.18	
	47.01	53.62	25.90	5.36	2.59	2.07	28.25	1.66	19.46	2.42	31.12	31.15
PCR	10.49	11.52	25.89	1.15	2.59	0.44	6.06	1.73	4.29	2.45	29.29	
	45.47	51.49	25.85	5.15	2.59	1.99	27.02	1.68	19.19	2.37	28.99	29.14
PCA	15.97	10.98	34.88	1.10	3.49	0.31	10.49	1.52	6.55	2.93	37.61	
	62.74	42.30	35.08	4.23	3.51	1.21	40.88	1.53	26.41	2.38	35.40	36.50

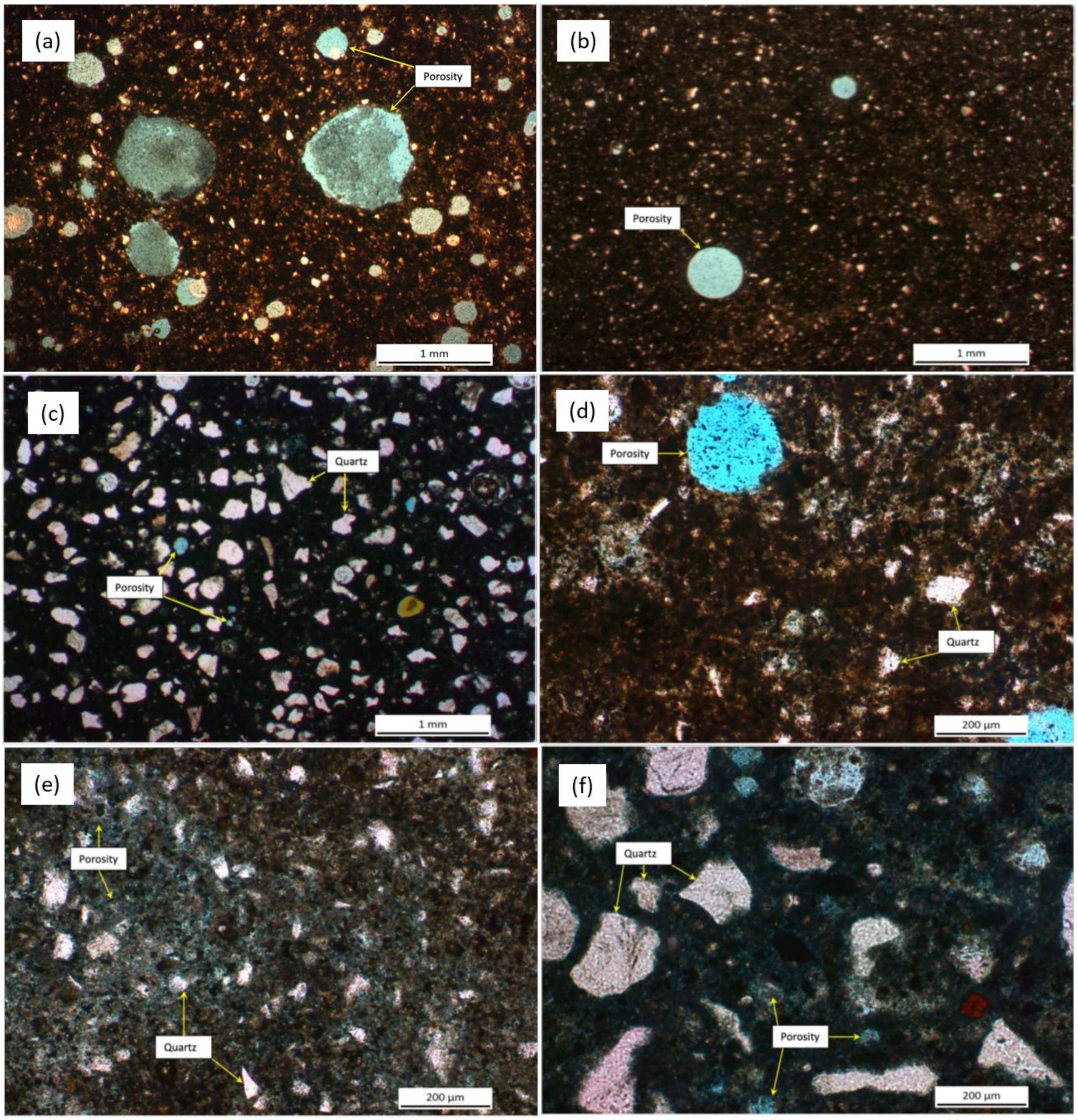

Figure S3. Plain polarised light optical microscope images of (a) PC, (b) PCR and (c) PCA at 2.5x magnification, and (d) PC, (e) PCR and (f) PCA at 10x magnification. All images taken before crushing for the experiments.

Supplementary Implications of Geological Hydrogen Storage

- **The Physical Implications of Hydrogen Storage**

This section will consider the physical implications that the wellbore endures during hydrogen injection and production (withdrawal). Both injection and production wells need to be suitable for intensive injection and production periods due to the cyclic nature of underground storage [27]. This changing pressure and stress state of the production system results in the possibility of erosion and corrosion of the wellbore. Erosion refers to the pure mechanical removal of material, and corrosion is defined as an electrochemical mechanism of material degradation [28]. Any erosion and/or corrosion that occurs during an operational lifetime can jeopardize the integrity of the components, materials, and flowlines. The main mechanism that can result in significant erosional damage of a wellbore in underground gas projects is liquid droplet erosion. This is solely found in 'wet gas' reservoirs, which pertains to the availability of water in a porous reservoir. When gas is being produced, water vapour can seep in during pressure change and some of this vapour can turn into droplets which can cause erosion. The rate of erosion is reliant on factors such as droplet size, gas velocity, gas density, impact velocity and frequency. The liquid droplet erosion is a particular problem in high velocity gas streams and when droplets are larger in size (>1 mm) [29]. To eliminate any potential erosion and/or corrosion, materials and production facilities are designed so that the flow velocity is below a calculated erosional velocity limit. This relates to a flow limit and speed that ensures safe operating conditions, but if the limit is exceeded, erosion and/or corrosion may arise. The erosional velocity limit is governed by the API RE 14E equation S1:

$$\mathcal{V} = c\ \sqrt{\rho} \qquad \text{(S1)}$$

This equation correlates erosional velocity ($\mathcal{V}$), where c is an empirical constant and ρ is the density. The empirical constant can be either 100 for 'continuous service', 125 for 'intermittent service' and 250 for when corrosion is not predicted.

Production casing and tube size is another factor to be considered to optimize injection and production and prevent erosion. A study conducted in a depleted gas field repurposed for natural gas storage in Dangang, China, calculated a minimum tubing diameter of 115 mm is required to prevent tubing erosion. A greater tubing diameter can be favourable for gas production as they can cope with higher production rates. Underground gas facilities in China utilise tubing diameters of 178 mm, in comparison to conventional gas wells with diameters of 150 mm [31]. However, the erosional velocity rate does change with increased casing and tubing size. Many limitations exist when calculating an erosional velocity limit for underground hydrogen storage, but many of the controlling erosional parameters are not known. The examples presented above concern the storage of natural gas, and although this can be used as an indication for hydrogen, hydrogen has different properties of density and viscosity. The lower values for density and viscosity of hydrogen mean the natural gas parameters are not representative for the storage of hydrogen. This raises the question of whether *hydrogen production rates are likely to contribute to enhanced water droplet erosion during injection and production?* The main method for limiting erosion and/or corrosion of steel equipment during the injection and production of gas from underground geological reservoirs, is to limit the flow velocity to an 'erosional velocity'. This limit is estimated upon the assumption that no erosion and/or corrosion would occur below it. The estimated limit of the erosional velocity for droplet erosion is 70-80 m/s. Hydrogen production and injection is expected to be much lower than this erosional limit and therefore, the production rates are not likely to contribute to enhanced water erosion.

Safety Measures

The safe and reliable operation of high-pressure, high-temperature batch reaction vessels was ensured through meticulous attention to experimental design and engineering principles. A crucial aspect of this approach involved utilizing 316 stainless steel, characterized by its high manganese content and low nickel concentration (<13%). This material selection effectively mitigated the vulnerability of the reaction vessels to degradation and blistering, thereby enhancing their overall performance and longevity. To fortify the structural integrity of the vessels, we employed 8 M12 x 35 mm high-tensile cap screws along with meticulously engineered o-ring seals. These measures facilitated an upper pressure limit of 65 MPa, guaranteeing operational stability even under demanding conditions. In order to maintain optimal functionality throughout the experiments, we exclusively employed high-pressure valves and instruments sourced from Top Industrie. Known for their superior quality and durability, these components exhibited exceptional tolerance and reliability, even when subjected to extreme conditions of up to 100 MPa. The potential risks associated with the rapid heating of the vessels due to the Joule-Thomson effect were proactively addressed. By carefully regulating the rate of hydrogen injection, we minimized the likelihood of hazardous thermal excursions. Moreover, as an additional layer of safety, we installed a hydrogen gas alarm system in the laboratory. This system comprised a Riken Keiki GD-A80 detector head equipped with an HW-6211 sensor and a GP-6001 single-channel monitor panel. This state-of-the-art technology was specifically chosen to promptly detect any potential hydrogen leaks, ensuring swift response and mitigating any associated risks.

- **REFERENCES**

1. van der Valk, K.; van Unen, M.; Brunner, L.; Gas, D., Inventory of risks associated with underground storage of compressed air (CAES) and hydrogen (UHS), and qualitative comparison of risks of UHS vs. underground storage of natural gas (UGS). **2020**.
2. Michanowicz, D. R.; Buonocore, J. J.; Rowland, S. T.; Konschnik, K. E.; Goho, S. A.; Bernstein, A. S., A national assessment of underground natural gas storage: identifying wells with designs likely vulnerable to a single-point-of-failure. *Environmental Research Letters* **2017,** *12*, (6), 064004.
3. OGA Well Strategy. https://www.nstauthority.co.uk/media/5947/oga-wells-strategy-2019.pdf. Assesses on 5th August 2022.
4. BGS Safe storage of hydrogen in porous rocks: the challenges and knowledge gaps. British Geological Society Press. https://www.bgs.ac.uk/news/safe-storage-of-hydrogen-in-porous-rocks-the-challenges-andknowledge-gaps/. Assessed on 5th August 2022.
5. Vrålstad, T.; Todorovic, J.; Saasen, A.; Godøy, R. In *Long-term integrity of well cements at downhole conditions*, SPE bergen one day seminar, 2016; OnePetro: 2016.
6. Laudet, J.-B.; Garnier, A.; Neuville, N.; Le Guen, Y.; Fourmaintraux, D.; Rafai, N.; Burlion, N.; Shao, J.-F., The behavior of oil well cement at downhole CO2 storage conditions: Static and dynamic laboratory experiments. *Energy Procedia* **2011,** *4*, 5251-5258.
7. Ajayi, T.; Gupta, I., A review of reactive transport modeling in wellbore integrity problems. *Journal of Petroleum Science and Engineering* **2019,** *175*, 785-803.
8. Yousuf, N.; Olayiwola, O.; Guo, B.; Liu, N., A comprehensive review on the loss of wellbore integrity due to cement failure and available remedial methods. *Journal of Petroleum Science and Engineering* **2021,** *207*, 109123.
9. Lupyana, S. D.; Maagi, M. T.; Gu, J., Common well cements and the mechanism of cement-formation bonding. *Reviews in Chemical Engineering* **2022,** *38*, (1), 17-34.
10. Rabia, H., *Well engineering & construction*. Entrac Consulting Limited London: 2002.
11. Omosebi, O.; Maheshwari, H.; Ahmed, R.; Shah, S.; Osisanya, S.; Hassani, S.; DeBruijn, G.; Cornell, W.; Simon, D., Degradation of well cement in HPHT acidic environment: Effects of CO2 concentration and pressure. *Cement and Concrete Composites* **2016,** *74*, 54-70.
12. Broni-Bediako, E.; Joel, O.; Ofori-Sarpong, G., Oil well cement additives: a review of the common types. *Oil Gas Res* **2016,** *2*, (1), 1-7.
13. Samsuri, A.; Junin, R.; Osman, A. M. In *The utilization of Malaysian local bentonite as an extender and free water controller in oil-well cement technology*, SPE Asia Pacific Oil and Gas Conference and Exhibition, 2001; OnePetro: 2001.
14. Wilcox, B.; Oyeneyin, B.; Islam, S. In *HPHT well integrity and cement failure*, SPE Nigeria Annual International Conference and Exhibition, 2016; OnePetro: 2016.
15. Kosmatka, S. H.; Panarese, W. C.; Kerkhoff, B., *Design and control of concrete mixtures*. Portland Cement Association Skokie, IL: 2002; Vol. 5420.
16. Fink, J., *Petroleum Engineer's Guide to Oil Field Chemicals and Fluids Ed. 2*. Elsevier Science: 2015; p 317-367.
17. Shi, Z.; Jessen, K.; Tsotsis, T. T., Impacts of the subsurface storage of natural gas and hydrogen mixtures. *International Journal of Hydrogen Energy* **2020,** *45*, (15), 8757-8773.
18. Viana, M.; Jouannin, P.; Pontier, C.; Chulia, D., About pycnometric density measurements. *Talanta* **2002,** *57*, (3), 583-593.
19. Nguyen, H. G. T.; Horn, J. C.; Bleakney, M.; Siderius, D. W.; Espinal, L., Understanding material characteristics through signature traits from helium pycnometry. *Langmuir* **2019,** *35*, (6), 2115-2122.
20. Eggertsson, G. H., *Constraining mechanical and permeability properties of the Krafla geothermal reservoir, North-East Iceland*. The University of Liverpool (United Kingdom): 2019.

21. Lamur, A.; Kendrick, J.; Eggertsson, G.; Wall, R.; Ashworth, J.; Lavallée, Y., The permeability of fractured rocks in pressurised volcanic and geothermal systems. *Scientific reports* **2017,** *7*, (1), 1-9.
22. Kuhinek, D.; Zoric, I.; Hrzenjak, P., Measurement uncertainty in testing of uniaxial compressive strength and deformability of rock samples. *Measurement Science Review* **2011,** *11*, (4), 112.
23. Holder, C. F.; Schaak, R. E., Tutorial on powder X-ray diffraction for characterizing nanoscale materials. In ACS Publications: 2019; Vol. 13, pp 7359-7365.
24. Bychkova, Y. V.; Nikolaeva, I. Y.; Ermina, O.; Tskhovrebova, A.; Shubin, I.; Stennikov, A., The details of a method for the preparation of solid geological samples for ICP-MS multielement analysis. *Moscow University Geology Bulletin* **2018,** *73*, (6), 520-526.
25. Grotti, M.; Todolí, J.-L., Nitric acid effect in inductively coupled plasma mass spectrometry: new insights on possible causes and correction. *Journal of Analytical Atomic Spectrometry* **2020,** *35*, (9), 1959-1968.
26. Cheng, K.; Zhu, D.-M., On calibration of pH meters. *Sensors* **2005,** *5*, (4), 209-219.
27. Wang, Y.; Zhang, J., An optimized calculation method of critical erosion flow rates of UGS injection/production wells. *Natural Gas Industry B* **2020,** *7*, (3), 262-268.
28. Sani, F. M.; Huizinga, S.; Esaklul, K.; Nesic, S., Review of the API RP 14E erosional velocity equation: Origin, applications, misuses, limitations and alternatives. *Wear* **2019,** *426*, 620-636.
29. Arabnejad, H.; Shirazi, S. A.; McLaury, B. S.; Shadley, J. R. In *A guideline to calculate erosional velocity due to liquid droplets for oil and gas industry*, SPE Annual Technical Conference and Exhibition, 2014; OnePetro: 2014.
30. Barton, N., Erosion in elbows in hydrocarbon production systems: Review document. **2003**.
31. Bai, M.; Shen, A.; Meng, L.; Zhu, J.; Song, K., Well completion issues for underground gas storage in oil and gas reservoirs in China. *Journal of Petroleum Science and Engineering* **2018,** *171*, 584-591.
